# De La Puberté Et De L’Age Critique Chez La Femme—Au Point De Vue Physiologique Hygienique Et Medicale, Et De La Ponte Periodique Chez La Femme Et Les Mammiféres, &c.

**Published:** 1844-08-10

**Authors:** 


### BIBLIOGRAPHICAL NOTICES.

*De la Puberté et de l'Age Critique Chez la Femme—au point de vue physiologique hygienique et medicale, et de la Ponte Periodique Chez la Femme et les Mammiféres, &c.* Par M. A. Raciborski, M. D., &c. Paris, 1844. 12mo., pp. 520.

Another novelty in Obstetrics—for it is true to say that this book belongs to midwifery: upon the great points of Obstetric Physiology is it founded; and though filled with rich practical details, useful to the physician, it is more particularly so to the obstetrician, whose ministry conducts him constantly to the treatment of female complaints.

Kerckring, Cruikshank, Lee, Pouchet, Negrier, Gendrin, and now Raciborski, leave no doubt that menstruation depends upon the periodical stated excitement of the reproductive organs coincident with the stated normal development of the Graafian vesicles, which as one ripens in each month, so it causes the woman to menstruate each month; for so great is the coincident excitement of the ovaries, the tubes and the womb, that they become intensely injected and red with the hyperæmia, which is relieved only by the effusion of the menstrual discharge.

This doctrine of menstruation is the more firmly established by a review of the progress in late years of micrographic science as applied to physiology. MM. Coste and Delpech, Rudolph Wagner, Schwann, Wharton Jones, Barry, and a host of other names, have made it clear that the yelk of the mammals is produced within the granular fluid of the Graafian vesicles; and this yelk, which grows rapidly in the last periods of its development, escapes from its cell as a spontaneous physiological act, once a month in the human female, and at fixed times and seasons in other genera of the mammalia. The *Ponte*, as M. Raciborski calls it, takes place regularly. The incubation, as perfected in the whole scene of gestation, depends upon other principles.

We heartily wish that some one who has leisure would translate this book and lay it bodily before our brethren. They would find it full of sound views and details as to the management of young persons approaching the period of their puberty—a period in which the complexion of their entire future is often given by the circumstances that usher them into the womanly estate. They would discover great good sense in the observations on the hygienic management of girls approaching their puberty,—of their alimentation—of their educational studies—of the means of increasing their *embonpoint*, or checking the too great development thereof. The clothing, the corset, exercises in dancing, swimming, riding, and intellectual education—all these topics are presented with a masterly and vigorous hand.

The volume ought to be printed here, were it only for the sake of getting into the hands of our brethren the remarks on Chlorosis, and particularly the discussions commencing at p. 251, of the value in chlorosis, of all the various chalybeates, which he divides into

1st *Division.*

| | |
|---|---|
| Substances not saline | Metallic. |
| | Protoxydised. |
| | Peroxydised. |

2d *Division.*

| | | |
|---|---|---|
| Compound saline substances | protoxydised | by a mineral acid, |
| | | by a vegetable acid, |
| | peroxydised | by a mineral acid, |
| | | by a vegetable acid. |

We have no space here for a full account of M. Raciborski's work, but we cannot refrain from noticing his remarks on iron reduced by means of hydrogen to the metallic state. This is effected by passing a stream of hydrogen gas over an oxide of iron heated to redness in a gun barrel, in which process the oxygen of the iron uniting with the hydrogen flies off as water, leaving the metal reduced, and impalpably fine—and which is beyond comparison with the best porphyrised iron. Messrs. Quevenne and Miquelard, who proposed this formula, assert that it comprises the two grand desiderata in the chalybeates, *very great activity* with *absolute insipidity.*

M. Raciborski gives a sufficiently clear exposé of the doctrines of Hæmatosis, which are so connected with many of the cases of chlorosis that the understanding of them appears indispensable to the well ordering of any system of measures designed to combat the menstrual affections in a state of disorder or suspension. M. R. treats from p. 325 to 358 the subject of the change of life in the female—a most important division of the work—and at p. 359 he commences his observations upon the spontaneous periodical *ponte* in women and mammals. He thinks that M. Pouchet is the first to lay down the fact that the spontaneous ovulation of the mammifera is a general law, and he does not attribute to M. Negrier of Angers so much merit on this point as in our opinion that gentleman may justly claim. But we shall extend this notice too far, if we suffer ourselves to be led into further temptation. We repeat, then, that we look upon it as a spirited and vigorous production, which could have been written only by a man of reflection, with high powers of mental analysis. M.

